# Alzheimer’s Disease Diagnostics Must Be Globally Accessible

**DOI:** 10.3233/JAD-210663

**Published:** 2021-12-07

**Authors:** Nicholas Clute-Reinig, Suman Jayadev, Kristoffer Rhoads, Anne-Laure Le Ny

**Affiliations:** a Global Health Labs, Bellevue, WA, USA; b Alzheimer’s Disease Research Center, Department of Neurology, The University of Washington, Seattle, WA, USA

**Keywords:** Alzheimer’s disease diagnostics, dementia, developing countries, global health, health equity, healthcare disparities, neurological diagnostic techniques

## Abstract

Dementia and Alzheimer’s disease (AD) are global health crises, with most affected individuals living in low- or middle-income countries. While research into diagnostics and therapeutics remains focused exclusively on high-income populations, recent technological breakthroughs suggest that low-cost AD diagnostics may soon be possible. However, as this disease shifts onto those with the least financial and structural ability to shoulder its burden, it is incumbent on high-income countries to develop accessible AD healthcare. We argue that there is a scientific and ethical mandate to develop low-cost diagnostics that will not only benefit patients in low-and middle-income countries but the AD field as a whole.

## DEMENTIA IN LOW- AND MIDDLE-INCOME COUNTRIES

As worldwide life expectancy increases, dementia has become one of the most wide-ranging and intractable global health issues. Currently the seventh most common cause of death worldwide [1], an estimated 50 million individuals live with dementia today—a number projected to triple by 2050 [2]. Though dementia was once considered a disease affecting high-income countries (HIC), approxi-mately 58%of cases are currently found in low- and middle-income countries (LMIC), and incidence appears to be stabilizing in HIC [2]. Thus, the burden of dementia is rapidly shifting toward LMIC.

Beyond the devastating human toll, the economic impact of dementia is estimated to cost above 1 trillion U.S. dollars globally, with roughly 18%of that amount in LMIC [3]. This disparity between the cost and prevalence of dementia in LMIC is in part due to lower wage costs and a higher proportion of informal care work—between 2010 and 2015, the dementia-related costs in LMIC increased by 63%; however, the amount spent on professional care did not change [3]. This suggests that as dementia incidence increases in LMIC there has not been a concomitant boost to the professional care economy, changes which would support diagnosis and management. Rather, it appears that a wave of economic burden affecting individual caregivers appears headed toward a vulnerable population.

## ACCESSIBLE ALZHEIMER’S DISEASE DIAGNOSTICS ARE REQUIRED TO MEET GOALS

To face this issue, the World Health Organization has proposed 50%of member countries to diagnose 50%of all cases of dementia by 2025 [1]. This goal is ambitious; dementia is widespread, while quality, scalable diagnostics are nascent. Nevertheless, diagnostic programs have been successfully implemented in many HIC with high enough fidelity to allow for the development and trials of diagnostics and therapeutics.

Though it is a complex and heterogeneous disorder, Alzheimer’s disease (AD) is the primary contributor to 60–70%of all dementia cases [1]; AD diagnostics must be a major part of reaching the World Health Organization goal. Diagnosing AD is a sophisticated clinical procedure involving cognitive assessment from a highly trained primary care or specialist healthcare provider. In addition, neuroimaging techniques and molecular biomarkers may assist in diagnosis, though these methods lack the scalability and accessibility needed for LMIC implementation [4]. While a reliable biomarker from an accessible patient sample would circumvent these issues, such methods have historically been prohibitively expensive, lacked scalability, or shown poor predictive value [5].

However, new research suggests high-fidelity, non-invasive testing is on the horizon. Plasma-based amyloid-β isoforms and phosphorylated tau biomarkers show efficacy in differentiating AD from other neurodegenerative diseases with near equivalence to neuroimaging techniques [6, 7]. Though these assays are still in development stages—and require instrumentation that renders them inaccessible to LMIC markets—they suggest that the development of low-cost plasma-based AD diagnostics may soon be possible.

## DEVELOPING LMIC DIAGNOSTICS WOULD TRANSFORM AD RESEARCH AND CARE

While technological advancement in AD diagnostics is on the horizon, there remain structural limitations to widespread diagnostic capacity in LMIC. Access to healthcare, availability of trained providers, poor validity of screening tests, and varied presentations of AD are all barriers to implementing a molecular diagnostic [8]. Yet, making high-fidelity diagnostics accessible to those living in LMIC is a crucial for goal, for humanitarian, social, and scientific reasons.

Though treatments for AD remain palliative, the benefit of early diagnosis in LMIC would be substantial. It would allow individuals to make plans for their care, giving them control over their futures and allowing for proactive community-based care as recommended by the WHO. Further, more readily performed diagnoses can reduce shame and fear around diagnosis, thereby decreasing social barriers to seeking care. Increased understanding also supports more extensive efforts to introduce lifestyle changes that may help manage risk factors for disease progression [8, 9]. One of the first steps in developing culturally appropriate dementia care capacity is to make diagnosis accessible regardless of patient resources.

Finally, an accessible high-fidelity diagnostic has the potential to significantly increase the patient pool for clinical trials, critically expanding the genetic and cultural backgrounds of test subjects. This would support one of the primary mandates for healthcare—addressing diversity and equity in health research and management—at a point when almost all clinical trials are conducted in HIC, particularly the U.S. and Europe. This lack of inclusion raises ethical concerns over designing care solely for a small handful of wealthy nations.

Given the resources required to find, diagnose, and enroll patients into trials, it is understandable that few trials include widespread diversity. Yet AD is a heterogeneous disorder with diverse genetic and environmental interactions which contribute to risk and presentation. Patient homogeneity poses a real scientific threat, not only to the applicability of limited trials, but to our basic understanding of AD etiology. Developing tools that are accessible outside the sphere of HIC healthcare would deepen our understanding of the disease, increase equity in disease management, and support the study of culture-specific palliative options, as a lack of diagnoses has led to few tested methods for care [10].

It is possible to integrate technological and structural changes to increase LMIC access to AD diagnostics. Funding mechanisms such as the NIH Fogarty International Center’s “Brain Disorders in The Developing World” program incentivize scientific collaboration between HIC and LMIC researchers while simultaneously developing diagnostic capacity in LMIC. If technological solutions are to work, it is imperative to increase the scope and funding of such dual-purpose, collaborative programs to combat structural barriers [8]. This extends to the private and philanthropic sectors, where industry and institutes should be incentivized to engage in technological development without ignoring structural limitations. Dementia remains one of the greatest global health challenges, but we believe that through the combined support of global health bodies, research institutions, and governmental funding mechanisms, diagnosis and care may be made accessible globally.

## DISCLOSURE STATEMENT

Authors’ disclosures available online (https://www.j-alz.com/manuscript-disclosures/21-0663r1).
